# The Prognostic Value of the Muscle Regional Oxygen Saturation Index in Patients with Acute Respiratory Distress Syndrome

**DOI:** 10.3390/jcm13247612

**Published:** 2024-12-13

**Authors:** Yen-Huey Chen, Kuo-Chin Kao, Meng-Jer Hsieh, Shaw-Woei Leu, Chung-Chi Huang

**Affiliations:** 1 Department of Respiratory Therapy, College of Medicine, Chang Gung University, Taoyuan 33301, Taiwan; yhchen@mail.cgu.edu.tw (Y.-H.C.); mengjer@cgmh.org.tw (M.-J.H.); 2 Division of Pulmonary and Critical Care Medicine, Chang Gung Memorial Hospital, Linkou. 5, Fu-Hsin St. Gweishan, Taoyuan 33353, Taiwan; swleu@cgmh.org.tw; 3 Department of Respiratory Therapy, Chang Gung University of Science and Technology, Chiayi 61363, Taiwan

**Keywords:** tissue oxygenation, near-infrared spectroscopy, acute respiratory distress syndrome

## Abstract

**Background**: Impaired systemic tissue oxygenation and microvascular perfusion are associated with adverse outcomes in patients with acute respiratory distress syndrome (ARDS). Tissue oxygenation and microvascular reactivity, assessed by using near-infrared spectroscopy (NIRS), are correlated with disease severity in critically ill populations. This study aimed to detect alterations in these factors and their ability to predict outcomes in patients with ARDS. **Methods**: We performed NIRS measurements on the first (Day 1) and third (Day 3) days after ARDS diagnosis in 29 patients. We recorded the baseline forearm muscle oxygen saturation (StO_2_) and calculated the deoxygenation slope (Deoxy) and reoxygenation (Reoxy) slope. We divided the subjects into 28-day survival and non-survival subgroups to compare microcirculatory and oxygenation status differences. **Results**: The Day 1 StO_2_ values were significantly higher for the survival subgroup (60.1 ± 13.5%) than the non-survival subgroup (47.2 ± 6.9%) (*p* = 0.025). The ROC curve showed that Day 1 StO_2_ was a significant predictor of 28-day mortality (*p* = 0.025). There was no significant difference between the Deoxy and Reoxy slopes of the two groups (*p* > 0.05). The ROC of the Day 1 Reoxy slope for survival prediction (AUC0.74) was not statistically significant (*p* = 0.074). **Conclusions**: Our study showed poor survival outcomes in patients who had lower skeletal muscle StO_2_ values in early-stage ARDS. NIRS measurements may provide prognostic value for the survival outcomes in patients with this syndrome.

## 1. Introduction

Acute respiratory distress syndrome (ARDS) is the most severe form of acute lung injury found in intensive care units. In it, bilateral dense and diffuse lung consolidation and collapse result in impaired gas exchange and severe refractory hypoxemia [1]. Although significant progress has been made in understanding the natural history and pathogenesis of this lethal syndrome, its attributable mortality has remained high and without improvement within recent years. Persistent and severe tissue hypoxia and multiple-organ failure are the leading causes of death. Approximately 10–15% of patients admitted to intensive care units and up to 23% of mechanically ventilated patients meet the criteria for ARDS [2]. In a review study, the overall mortality rate for the ARDS population has been reported to be about 43%. The mortality for ARDS is proportional to the severity of the disease: 45%, 32%, and 27% for severe, moderate, and mild disease, respectively [3,4]. Early detection of the onset of ARDS and providing appropriate management are very important for the affected population.

ARDS often starts within seven days of the inciting event and is characterized by bilateral lung infiltrates and severe progressive hypoxemia in the absence of any evidence of cardiogenic pulmonary edema [5]. In addition to systemic hypoxia, endothelial and epithelial impairment affect patients’ microcirculation and hospitalization outcomes [6]. Studies have demonstrated that microcirculation abnormalities are related to multiple-organ dysfunction and poor outcomes in critically ill patients [7,8]. Trzeciak et al. assessed the microcirculation function in patients with severe sepsis/septic shock and reported that the microcirculatory indices were more severely impaired in non-survivors compared with surviving patients [9].

Near-infrared spectroscopy (NIRS) is a tool used for monitoring tissue oxygen saturation (StO_2_) and microvascular dysfunction in acutely ill patients [10]. It involves three principal measurements: (1) StO_2_; (2) the StO_2_ deoxygenation slope (Deoxy slope); and (3) the StO_2_ recovery slope (Reoxy slope) in response to the vascular occlusion test (VOT) [11,12]. StO_2_ is a marker of tissue perfusion and oxygenation status depending on the device used and location of measurement [13,14]. Previous studies have reported an association between hypoperfusion and tissue oxygen saturation [15,16,17]. Skarda et al. performed NIRS measurements in healthy volunteers and patients with severe sepsis and observed lower StO_2_ values in the latter compared with the former [17].

The vascular occlusion test (VOT) is a provocative test in which StO_2_ is measured on a distal site (the thenar eminence or forearm), while transient rapid vascular occlusion is performed for either a defined time interval or until the StO_2_ decreases to a defined minimal threshold (the Deoxy slope). The pressure is then released, allowing blood flow to return to the baseline (Reoxy slope). The Reoxy slope is considered as an indicator of reactive hyperemic response [18]. Reactive hyperemia indicates microcirculatory reactivity, which suggests the tissue’s ability to deliver oxygen after a hypoxic stimulus induced by a transient interruption in blood flow. Creteur et al. have demonstrated that StO_2_ recovery slopes were lower in patients with sepsis and that the occurrence of this alteration in the first 24 h of sepsis was associated with a worse outcome [19].

Many NIRS studies have been performed on sepsis, trauma, and heart failure patients. However, only some have examined the utilization of NIRS in assessing microcirculation and tissue oxygenation status in patients with ARDS. This study aimed to assess tissue oxygenation and microcirculation statuses via static and dynamic NIRS-derived variables and further examine their prognostic value in ARDS outcomes.

## 2. Materials and Methods

### 2.1. Study Design and Participants

This was a prospective single-center observational study conducted in the medical intensive care unit (ICU) of CHANG GUNG MEMORIAL HOSPITAL, Linko, Taiwan. We included mechanically ventilated patients with ARDS conforming to the Berlin Definition of Acute Respiratory Distress Syndrome [20]. The ARDS criteria are as follows: (1) timing: within one week of a known clinical insult or new or worsening respiratory symptoms; (2) chest radiology: bilateral infiltration not fully explained by effusions, lobar/lung collapse, or nodules; (3) origin of edema: respiratory failure not fully explained by cardiac failure or fluid overload requiring an objective assessment (e.g., echocardiography) to exclude hydrostatic edema if no risk factors are present; and (4) oxygenation: at least mild ARDS with an arterial oxygen partial pressure/fraction of inspired oxygen (PaO_2_/FiO_2_) of >200 mmHg and ≤300 mmHg, with positive end-expiratory pressure (PEEP) or continuous positive airway pressure (CPAP) of ≥5 cm H_2_O of mechanical ventilators [20]. We also included patients with stable hemodynamic status mean arterial pressures (MAPs) of >60 mmHg with no change in their vasopressor infusion rate for two hours (Table 1).

The exclusion criteria were age of <18 years, pregnancy, established “Do Not Resuscitate” orders before enrollment, any acute cerebrovascular events (primary diagnosis), acute coronary syndrome with myocardial infarction (primary diagnosis), acute and active gastrointestinal bleeding (primary diagnosis), acute drug overdose (primary diagnosis), and the requirement for extracorporeal membrane oxygen (ECMO) support (Table 1). This study was approved by the hospital’s institutional review board (IRB No.: 201902078B0; date of approval: 6 January 2020) and was performed in accordance with the Declaration of Helsinki. We obtained written informed consent from the participants or their relatives. We recorded participants’ age, gender, reason for ICU admission, comorbidities, and acute physiology and chronic health evaluation (APACHE) II scores at enrollment.

### 2.2. Study Procedures

After ICU admission and hemodynamic stabilization (mean arterial pressure (MAP) of >60 mmHg with no change in vasopressor infusion rate for two hours), we recorded the global hemodynamic variables (including cardiac output, systolic and mean arterial pressures, and stroke volume) using a PiCCOplus monitor (version 5.2.2; Pulsion Medical Systems AG, Stahlgruberring Munchen). The same day, we performed an NIRS measurement that included a vascular occlusion test. We measured muscular StO_2_ for one minute to represent the baseline StO_2_ after five minutes of stabilization. We then performed a vascular occlusion test to calculate deoxygenation StO_2_ slopes. We collected data on the day of ICU admission (Day 1) and at 48 h (±2 h) (Day 3). We followed the subjects until they were discharged from the ICU. We recorded outcomes such as the duration of mechanical ventilation, duration of ICU stay and hospital stay for survivors, and ICU and in-hospital mortality rates.

### 2.3. Measurements

#### 2.3.1. Hemodynamic Status Measurement

We recorded heart rates (HRs) and blood pressure with an online HP Component Monitoring System (Model 56S, M 1165A; Hewlett-Packard, Boblingen, Germany). A 4F thermistor-tipped arterial catheter (Pulsiocath; Pulsion Medical Systems) was inserted into the femoral artery and connected to a bedside hemodynamic PiCCOplus monitor (version 5.2.2; Pulsion Medical Systems AG, Stahlgruberring Munchen, Germany). The continuous pulse contour cardiac output and pulse contour stroke volume were measured using the heart rate and the area under the aortic flow curve.

#### 2.3.2. Near-Infrared Spectroscopy Assessment and Vascular Occlusion Test

**Figure 1:**
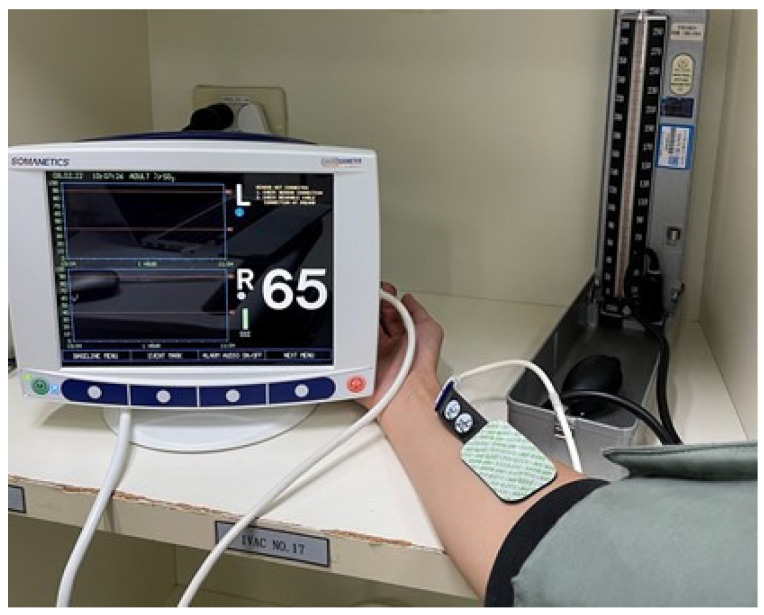
NIRS device (with value of StO_2_ (65%) in skeletal muscle).

**Figure 2:**
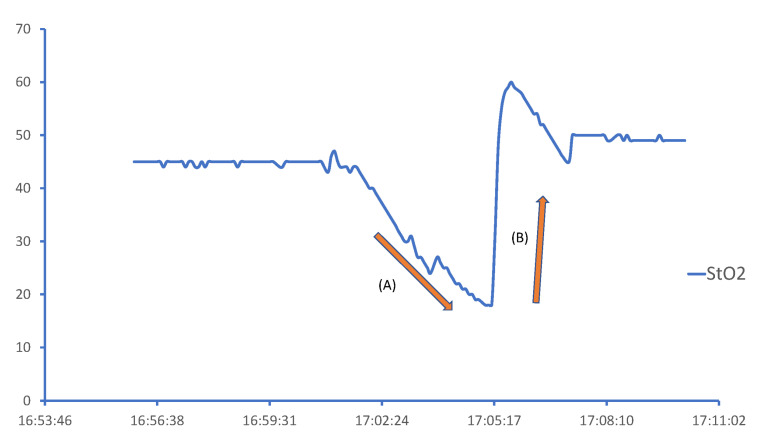
Changes in StO_2_ in skeletal muscle during vascular occlusion test (VOT). (A) Deoxy slope; (B) Reoxy slope.

We performed muscular StO_2_ measurements using a commercially available NIRS system with nonsterile, noninvasive, disposable skin-surface probes (INVOS 5100C Oximeter and adult SomaSensor model SAFB-SM; Somanetics Corporation, Troy, MI, USA) placed on the medial forearm at 5 cm distal to the elbow and on the middle thigh (Figure 1). Following a five-minute stabilization period, we performed the initial StO_2_ measurement and then a VOT procedure using a sphygmomanometer around the upper arm. The sphygmomanometer was rapidly inflated to 50 mmHg more than the systolic pressure, which was maintained until the StO_2_ decreased to 40%. After the ischemic period, the sphygmomanometer was rapidly deflated, and we followed the StO_2_ response until it returned to baseline (Figure 2). We imported the StO_2_ measurements into a Microsoft Excel software file and derived the slopes by drawing a best-fit line for each metric’s steady-state slope. We calculated the StO_2_ occlusion; the steady-state rate of occlusion (StO_2_%/second), represented by the descending slope during the ischemic period (the Deoxy slope); and StO_2_ recovery, represented by the steady-state ascending slope during the reoxygenation phase after the cuff pressure was released (the Reoxy slope).

### 2.4. Statistical Analysis

The analysis was performed using SPSS for Windows (v22; IBM Co., Chicago, IL, USA). Normality was analyzed with the Shapiro–Wilk test. Discrete variables were expressed as counts (percentage) and continuous variables as means ± standard deviations. For the subjects’ demographic and clinical characteristics, we assessed the differences between the groups using the Chari-Squared test and Fisher’s exact test for the categorical variables and the Student *t*-test, Mann–Whitney U test, or Kruskal–Wallis test for the continuous variables. The predictive values for the resuscitation variables on the outcome variables were analyzed by using a receiver operator characteristic (ROC) curve. We computed the area under the ROC curve and Youden’s index to identify the best cutoff value. A *p*-value of less than 0.05 indicated statistical significance.

## 3. Results

We enrolled 29 subjects with ARDS, including 24 males and 5 females, with a mean age of 60 ± 15.6 years old. Table 1 shows the clinical characteristics of the enrolled patients. The mean SOFA and APACHE II scores were 10.3 ± 4.2 and 24.4 ± 5.9, respectively. The mean P/F ratio was 130.9 ± 64.2%. Regarding ARDS severity, 5 subjects (17.2%) were categorized as mild, 13 subjects (44.8%) were categorized as moderate, and 11 (37.9%) subjects were categorized as severe (Table 2). During the 28-day follow-up, twenty-two subjects remained alive (survivor group) and seven subjects expired (non-survivor group) due to multiple-organ failure (*n* = 3) and septic shock (*n* = 4).

In the hemodynamic measurements, there were no significant differences in the Day 1 cardiac index, stroke volume index, or blood pressure between survivors and non-survivors (Table 3). In the NIRS measurements, the Day 1 forearm StO_2_ value was significantly higher for the survival group than the non-survival group (60.1 ± 13.5% vs. 47.2 ± 6.9%, *p* = 0.025) (Table 3). The reoxygenation slope in the survivors was higher than that in the non-survivors; however, the difference was not statistically significant (*p* = 0.074) (Table 3). There were no significant differences between the groups regarding the Day 3 NIRS parameter measurements.

**Figure 3:**
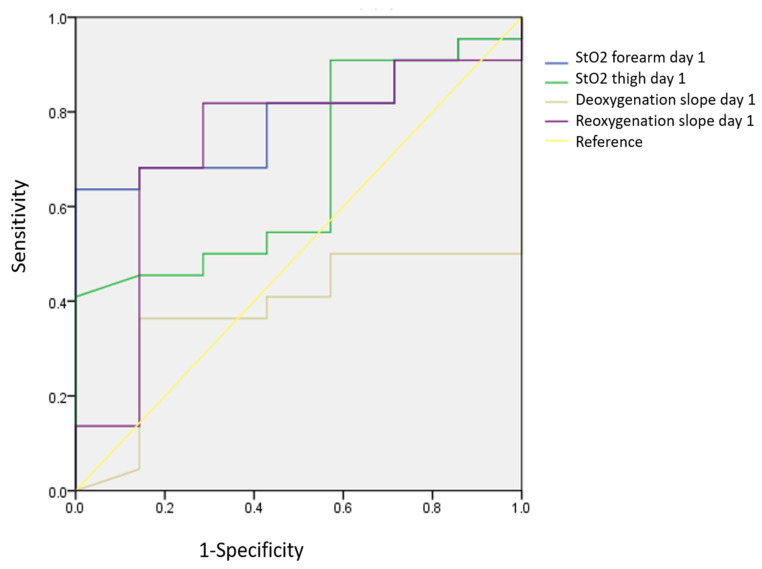
ROC curves of Day 1 NIRS parameters for 28-day survival.

**Figure 4:**
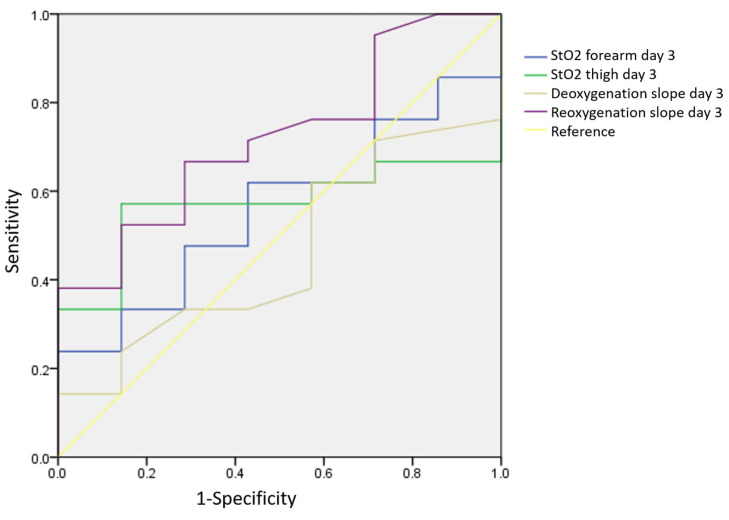
ROC curve of Day 3 NIRS parameters for 28-day survival.

The ROC curve analysis for the NIRS parameters revealed that Day 1 forearm StO_2_ has good predictability for 28-day survival in patients with ARDS, with an area under the ROC curve of 0.786 (*p* = 0.025), a sensitivity of 63.6%, and a specificity of 100% (Figure 3). A forearm StO_2_ value higher than 56.2% at Day 1 predicted the status of survival after 28 days. The Day 1 reoxygenation slope (cutoff point: 1.9%/s) was less predictive of 28-day survival than the StO_2_ slope (ROC AUC of the reoxygenation slope: 0.740, *p* = 0.074) (Figure 3). None of the Day 3 NIRS parameters significantly predicted 28-day survival (Figure 4).

Table 4 lists the ROC AUC, sensitivity, specificity, 95% confidence interval, and cutoff value of each NIRS parameter (the StO_2_ of the forearm and thigh and the reoxygenation slope) of the 28-day survivors and non-survivors.

**Figure 5:**
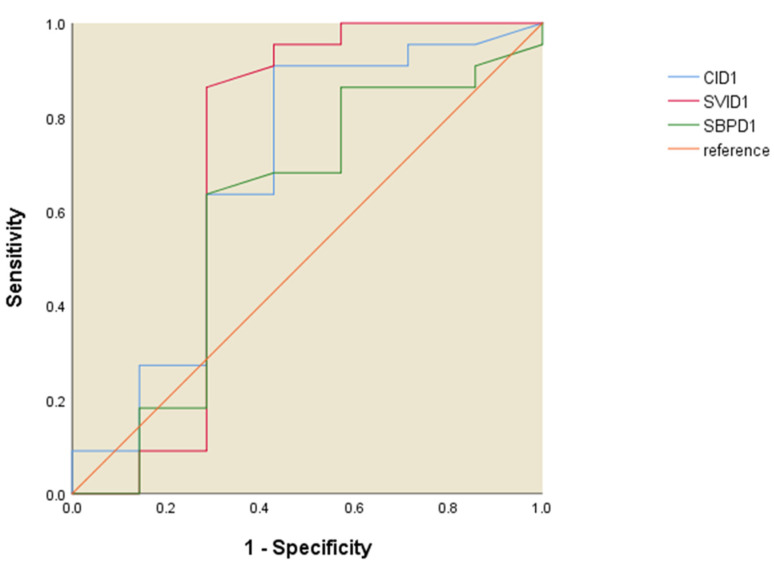
ROC curve of Day 1 hemodynamic parameters for 28-day survival. (CI: cardiac index; SVI: stroke volume index; SBP: systolic blood pressure. D1: Day 1).

**Figure 6:**
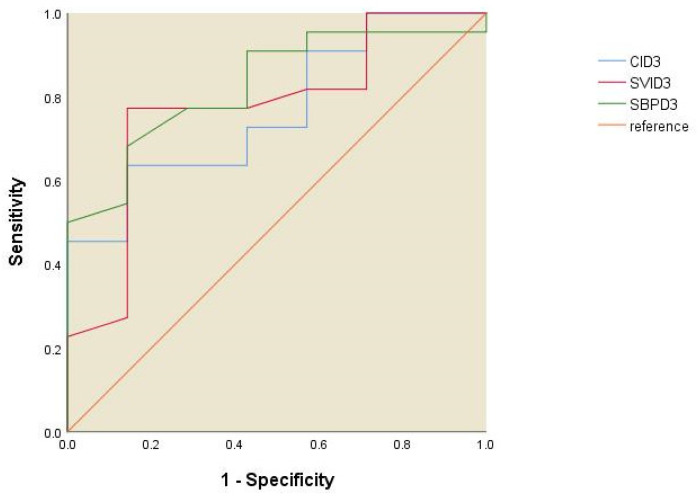
ROC curve of Day 3 hemodynamic parameters for 28-day survival. (CI: cardiac index; SVI: stroke volume index; SBP: systolic blood pressure. D3: Day 3).

Table 5 lists the AUC, sensitivity, specificity, and cutoff values of hemodynamic parameters of the survivors and non-survivors. None of these hemodynamics parameters at Day 1 significantly predicted 28-day survival (Table 5, Figure 5). At Day 3, the ROC curve analysis for the hemodynamic parameters revealed that cardiac index (CI), stroke volume index (SVI), and systolic blood pressure have good predictability for 28-day survival in patients with ARDS (Figure 6). At Day 3, an SBP higher than 118.5 mmHg (*p* = 0.010), SVI higher than 37 mL/m^2^ (*p* = 0.032), or CI higher than 3.62 L/min/m^2^ (*p* = 0.037) predicted the status of survival after 28 days (Table 5, Figure 6). 

## 4. Discussion

Our study demonstrated a significantly lower NIRS-derived static parameter of forearm StO_2_ on Day 1 for the non-survivors than the survivors. The forearm StO_2_ on Day 1 also predicted the 28-day survival outcomes. The NIRS-derived dynamic parameters obtained during the VOT did not differ between the non-survivors and survivors.

NIRS has been used to assess disease severity and survival outcomes in various populations. Tamura et al. assessed StO_2_ during cardiopulmonary bypass in patients who underwent cardiac surgery and reported that it was lower in patients with postoperative lung complications than those without them [21]. Filho et al. examined the peripheral perfusion function of patients admitted to the ICU with and without circulatory shock and reported that the former had significantly lower StO_2_ values than the latter [22]. Cortes et al. reported that patients with ARDS had lower StO_2_ values than healthy adults. Individuals with ARDS who required fewer than seven days of ventilatory support (good evolution) had higher StO_2_ values than those who required it for more than seven days or died [23]. In our study, the StO_2_ on the first day after an ARDS diagnosis was significantly lower in the non-survivors than the survivors. The StO_2_ obtained from the NIRS indicated tissue oxygenation utilization and delivery. The decreases in StO_2_ in those with ARDS may indicate impaired systemic tissue oxygenation.

We also found that the forearm StO_2_ on Day 1 had good predictability for 28-day survival, with an ROC AUC of 0.786. Marin-Corral et al. reported that the StO_2_ 24 h after the onset of septic shock in their studied patients was a good mortality predictor, with an AUC of 0.79 [24]. Macdonald’s cohort study found that StO_2_ was associated with in-hospital mortality, with an ROC AUC of 0.77 [25]. Tamura et al. found that the StO_2_ during cardiopulmonary bypass significantly predicted postoperative pulmonary complications in their studied patients, with an AUC of 0.71 [21]. Adequate tissue oxygenation is essential for proper organ function and improved survival outcomes. ARDS is a severe respiratory failure in which alveolar damage, inflammation, and pulmonary fluid accumulation impair gas exchange and contribute to hypoxemia [1], which leads to systemic hypoxia, affecting organ and peripheral tissue oxygenation status. Previous studies have reported the prognostic value of skeletal muscle StO_2_ in patients with sepsis, trauma, and circulatory problems [21,25,26]. This StO_2_ reflects the balance between local oxygen supply and consumption [27]. Lower StO_2_ may result from reduced O_2_ extraction or higher O_2_ consumption, which have been associated with worse outcomes. In critical care, it is important to recognize patients at risk of poor prognosis. Our findings are consistent with those of previous studies suggesting that this NIRS-derived parameter may help predict outcomes in patients with compromised tissue oxygenation.

In a study examining the relationship between organ failure and microcirculation, trauma patients, who have significantly more impaired microvascular reactivity on the first day of ICU admission, had more severe organ dysfunction at the end of their ICU stay. There were no differences in microvascular reactivity on Day 3 and Day 4 between the high- and low-severity groups [28]. Our study found a significant difference in the NIRS-derived parameters between the survivors and non-survivors on Day 1 but not Day 3. These early measurements reflect the initial systemic response to acute ARDS, which may be a critical determinant of outcomes. The Day 1 StO_2_ was significantly lower in the non-survivors than the survivors, indicating a lower tissue oxygenation status. However, there was no difference in the Day 3 measurement between the groups. Lower tissue oxygenation may impact organ dysfunction and patient health outcomes. Maintaining adequate tissue oxygenation in the early stage of ARDS could promote better physiological reserves in those with it. In addition, the Day 3 StO_2_ survival predictabilities were lower and less significant than on Day 1. By Day 3, patients could have undergone various therapeutic interventions, such as medication, mechanical ventilation, or fluid management, which could alter tissue oxygenation status, making it less reliable for predicting survival outcomes.

The dynamic parameters derived from the VOT depend on microvascular reactivity and the ability of tissues to respond to ischemic challenges. We obtained the deoxygenation slope (Deoxy) during the VOT, which indicated the velocity of the decrease in the StO_2_ during ischemia. Deoxy reflects local aerobic metabolism, including oxygen consumption. We also obtained the reoxygenation slope (Reoxy), which is the velocity of StO_2_ recovery following the VOT. Reoxy reflects microvascular reactivity and microcirculation function [24]. This dynamic NIRS-derived parameter obtained during the VOT can detect microvascular dysfunction in patients with critical illness. In a study of patients in the ICU with sepsis, respiratory disease, and trauma, Donati et al. reported that the Reoxy slopes in non-survivors were lower than in survivors, suggesting a reduction in microvascular reactivity and tissue perfusion in this population. They also reported that the Reoxy slope on the day of ICU admission predicted 90-day mortality, with an ROC AUC of 0.68 [29]. In our study, the reoxygenation slope for the survivors was higher than that for the non-survivors. We also found that the ROC AUC of the Reoxy slope for predicting the 28-day survival status was 0.727. Factors such as inflammatory mediators and tissue hypoxemia, which are common in ARDS, could affect vasodilation in the post-occlusive reactive hyperemia stage [1,2,3,23]. Microvascular function impairment may lead to multiple-organ failure and poor prognosis. Further studies with larger sample sizes are required to examine this mechanism further.

The early prognostication of ARDS outcomes is challenging. Tissue oxygenation impairment is an essential component of the pathogenesis of this syndrome. Monitoring muscle oxygenation using near-infrared spectroscopy clarifies the systemic oxygenation and perfusion status in it. This supports the early detection of hypoxia, guides therapeutic interventions, and serves as a prognostic indicator of outcomes. NIRS is a noninvasive and easily repeatable bedside test. Our study suggests that an NIRS measurement the day after an ARDS diagnosis would help to identify a patient at a high risk of a poor outcome, potentially creating a target population for early intervention tests.

In our study, the Day 1 forearm StO_2_ was significantly higher in the survival group than in the non-survival group, whereas no significant difference was found in Day 1 hemodynamics variables. However, at Day 3, hemodynamic variables (CI, SVI, and SBP) were significantly higher in the survival group than in the non-survival group, whereas no difference was found in Day 3 StO_2_. The phenomenon where microcirculatory function is impaired despite systemic hemodynamic variables remaining stable has been reported in patients with critical illness [8,30]. Hernandez et al. observed that the alteration of microcirculation function was related to mortality in septic shock patients, whereas no relationship was found between microcirculation and hemodynamic parameters [30]. Backer et al. reported that hemodynamic variables (mean arterial pressure and cardiac index) were relatively preserved at Day 1 and Day 2, whereas the microcirculatory variables were significantly altered in patients with critical illness [8]. The causes for the heterogeneity between microcirculation and microcirculation could be multifactorial. ARDS is often associated with the release of inflammatory mediators (e.g., IL6 and TNF-α) [31]; reactive oxidative species [31] that may disrupt endothelial and vascular integrity, causing capillary leakage; decreased microvascular flow; and compromised vascular regulation, leading to tissue hypoperfusion. Oxygen supply and utilization at the tissue level is limited [31,32,33]. In this case, the alterations in microvascular perfusion persist even when the systemic hemodynamic status was corrected [34], and the severity is associated with a poor outcome [34].

### Limitations

This study had several limitations. First, the sample size was relatively small. Also, we did not adjust the correlations between the NIRS parameters and the disease severity scores for confounders. The characteristics and treatment varied among the patients, which may have contributed to heterogeneity. Therefore, some analyses may be underpowered. Second, as this was an observational study, we could not determine the causal relationship between improvements in tissue oxygenation and outcomes. Interventional studies incorporating NIRS parameters among resuscitation targets may be required to assess the casual relationships between increased tissue oxygenation, microvascular function, and better outcomes. Third, we did not record oxygen content status measurements such as SaO_2_ and hemoglobin values; it was difficult to discriminate if changes in STO_2_ are related to oxygen content changes. Fourth, previous studies have measured skeletal muscle StO_2_ with different devices and methodologies, making it difficult to compare our parameters with those studies. For example, in a study examining the microcirculation of patients with ARDS, the mean StO_2_ was 75% [23], whereas it was 57% in our study. However, both studies found significant differences in StO_2_ values between survivors and non-survivors. This suggests that StO_2_ has prognostic implications for patients with ARDS with impaired oxygenation function.

## 5. Conclusions

Our study showed that patients with ARDS who have lower skeletal muscle StO_2_ values on their first day in the ICU have a poor prognosis during their hospitalization. The lower StO_2_ may indicate more severe impairment in systemic tissue oxygenation in the progression of ARDS and contribute to worse outcomes. The deoxygenation and reoxygenation slopes during the vascular occlusion test (VOT), however, were not significantly different between the survivors and non-survivors. The application of NIRS may provide prognostic value for assessing ARDS outcomes. However, further research is warranted to substantiate the potential benefits of evaluating skeletal muscle StO_2_ in patients with ARDS.

## Figures and Tables

**Table 1:** Inclusion and exclusion criteria of subjects.

Inclusion Criteria	Exclusion Criteria
Mechanical ventilation and diagnosis of ARDS; Stable hemodynamic status.	Age < 18 years; Pregnancy; Established “Do Not Resuscitate” orders before enrollment; Acute cerebrovascular event (primary diagnosis); Acute coronary syndrome with myocardial infarction (primary diagnosis); Acute and active gastrointestinal bleeding (primary diagnosis); Acute drug overdose (primary diagnosis); Requirement of ECMO support.

**Table 2:** Basic characteristics of participants.

	All	Survivors (*n* = 22)	Non-Survivors (*n* = 7)	*p*
Age (years)	60 ± 15.6	60 ± 15.1	59 ± 15.4	0.705
Sex (M/F)	24/5	21/1	3/4	
Body height (cm)	166.1 ± 13.6	169 ± 5.9	157.4 ± 59.3	0.003
Body weight (kg)	61.1 ± 19.2	64.3 ± 9.1	50.9 ± 21.3	0.004
BMI (kg/m^2^)	22.1 ± 6.4	22.6 ± 3.3	20.5 ± 1.8	0.114
SOFA score	10.3 ± 4.2	9.7 ± 4.0	12.3 ± 4.1	0.960
APACHE score	24.2 ± 5.9	22.6 ± 4.9	28.7 ± 3.3	0.306
PaO_2_ (mmHg)	87.2 ± 37.5	87.9 ± 40	84.7 ± 23.4	0.559
P/F ratio	130.9 ± 64.2	129.9 ± 64.8	134.4 ± 27.3	0.264
ARDS severity				0.751
Mild (*n*)	5	4	1	
Moderate (*n*)	13	9	4	
Severe (*n*)	11	9	2	

BMI: body mass index; SOFA score: sequential organ failure assessment score; APACHE score: acute physiology and chronic health evaluation score; P/F ratio: PaO_2_/FiO_2_; ARDS: acute respiratory distress syndrome.

**Table 3:** Comparisons of Day 1 and Day 3 parameters between survivors and non-survivors.

	All	Survivor (n = 22)	Non-Survivor (n = 7)	*p*	Survivor (n = 22)	Non-Survivor (n = 7)	*p*
	Day 1	Day 1	Day 1		Day 3	Day 3	
CI (l/min/m^2^)	3.54 ± 0.97	3.7 ± 0.91	3.05 ± 1.06	0.161	3.88 ± 0.80	2.90 ± 1.09	0.037
SVI (ml/m^2^)	38.44 ± 9.47	39.9 ± 7.13	33.8 ± 14.4	0.108	41.47 ± 8.62	30.80 ± 10.93	0.032
SVRI (DS m^2^/cm^5^)	1662.9 ± 460.9	1623.1 ± 425.6	1787.9 ± 577.2	0.445	1758.9 ± 438.9	2190.1 ± 1188.6	0.919
SBP (mmHg)	118 ± 21	119 ± 19	116 ± 27	0.444	132 ± 20	109 ± 11	0.01
DBP (mmHg)	62 ± 13	63 ± 11	59 ± 18	0.371	70 ± 14.9	59 ± 11	0.097
Heart rate (bpm)	96 ± 17	94 ± 16	105 ± 19	0.272	95 ± 17	93 ± 16	0.919
StO_2_, forearm (%)	57.0 ± 13.7	60.1 ± 13.5	47.2 ± 6.9	0.025	59.6 ± 13.62	57.6 ± 9.63	0.652
StO_2_, thigh (%)	64.4 ± 11.9	66.0 ± 12.6	59.6 ± 8.0	0.177	65.3 ± 21.32	63.5 ± 5.04	0.577
Deoxygenation slope (%/sec)	0.14 ± 0.07	0.13 ± 0.06	0.11 ± 0.04	0.235	0.02 ± 0.54	0.07 ± 0.04	0.648
Reoxygenation slope (%/sec)	1.56 ± 0.85	1.7 ± 0.85	1.1 ± 0.71	0.074	1.25 ± 0.80	0.68 ± 0.32	0.084

CI: cardiac index; SVI: stroke volume index; SVRI: systemic vascular resistance index; SBP: systolic blood pressure; DBP: diastolic blood pressure; StO_2_: tissue oxygen saturation index.

**Table 4:** Sensitivities, specificities, and cutoff point of NIRS for Day 1 and Day 3 parameters of 28-day survival.

	StO_2_ Forearm		StO_2_ Thigh		Reoxygenation Slope	
	Day 1	Day 3	Day 1	Day 3	Day 1	Day 3
Cutoff	56.2%	72%	66.4%	66.4%	1.49%	79%
Sensitivity (%)	63.6	23.8%	57.1%	57.1%	68.2%	66.7%
Specificity (%)	100%	100%	85.7%	85.7%	85.7%	71.4%
ROC curve	0.786	0.558	0.571	0.571	0.740	0.721
95% CI	(0.622–0.949)	(0.336–0.779)	(0.365–0.778)	(0.365–0.778)	(0.545–0.924)	(0.518–0.873)
*p*	0.025	0.652	0.577	0.577	0.074	0.085

ROC: receiver operator characteristic; CI: confidence interval; StO_2_: tissue oxygen saturation index.

**Table 5:** Sensitivities, specificities, and cutoff points of hemodynamics parameters for Day 1 and Day 3 parameters of 28-day survival.

	CI (L/min/m^2^)		SVI (mL/m^2^)		SBP (mmHg)	
	Day 1	Day 3	Day 1	Day 3	Day 1	Day 3
Cutoff	2.57	3.62	33.2	37.0	112.5	118.5
Sensitivity (%)	0.909	0.636	0.864	0.773	0.636	0.682
Specificity (%)	0.571	0.857	0.714	0.857	0.714	0.857
ROC curve	0.679	0.766	0.705	0.773	0.597	0.828
95% CI	0.413–0.944	0.580–0.953	0.391–1.00	0.567–0.978	0.324–0.871	0.669–0.986
*p*	0.161	0.037	0.108	0.032	0.445	0.010

CI: cardiac index; SVI: stroke volume index; SBP: systolic blood pressure; ROC: receiver operator characteristic; CI: confidence interval; StO_2_: tissue oxygen saturation index.

## Data Availability

Data are available from the first author upon reasonable request.
